# A molecular and ecological study of *Grillotia* (Cestoda: Trypanorhyncha) larval infection in small to mid‐sized benthonic sharks in the Gulf of Naples, Mediterranean Sea

**DOI:** 10.1002/ece3.7933

**Published:** 2021-09-18

**Authors:** Mario Santoro, Bruno Bellisario, Fabio Crocetta, Barbara Degli Uberti, Marialetizia Palomba

**Affiliations:** ^1^ Department of Integrative Marine Ecology Stazione Zoologica Anton Dohrn Naples Italy; ^2^ Department of Agricultural and Forestry Sciences (DAFNE) University of Viterbo Viterbo Italy; ^3^ Department of Animal Health Istituto Zooprofilattico Sperimentale del Mezzogiorno Portici Italy; ^4^ Department of Public Health and Infectious Diseases Section of Parasitology Sapienza University of Rome Rome Italy

**Keywords:** *Dalatias licha*, *Etmopterus spinax*, *Galeus melastomus*, *Grillotia* sp., marine food webs, Mediterranean Sea, parasite infection, *Scyliorhinus canicula*, Trypanorhyncha

## Abstract

**Aim:**

Trypanorhyncha cestodes comprise a wide range of heteroxenous parasites infecting elasmobranchs as definitive hosts. Limited data exist on the larval infection of these cestodes and the role of intermediate and paratenic hosts in the life cycle of these parasites. We investigated the factors that determine the occurrence and the level of infection of *Grillotia* plerocerci in the skeletal muscles of various benthonic sharks and analyzed the parasites through an integrative taxonomic approach.

**Location:**

Mediterranean Sea.

**Methods:**

Sharks obtained as bycatch of commercial trawling activities (i.e., *Etmopterus spinax*, *Galeus melastomus*, and *Scyliorhinus canicula*) were used in this study. Data from a limited number of *Dalatias licha* and *Scyliorhinus stellaris* were also included. *Grillotia* plerocerci were molecularly characterized using the partial 28S large subunit rDNA. Boosted regression trees were used to model the relationship between the abundance of infection with both morphological and physiological predictors in each host.

**Results:**

Plerocerci of *Grillotia* were detected in all shark species except *S. stellaris*. Host species significantly differed in terms of parasite abundance, with the highest and lowest prevalence and abundance of infection detected in *G. melastomus* and *E. spinax*, respectively. The relative influence of the traits involved in explaining the parasite abundance was related to the host size in *G*. *melastomus*, while both morphology‐ and physiology‐related traits explained the patterns observed in *E*. *spinax* and *S*. *canicula*. The 28S rDNA sequences shared an identity of ∼99.40% with a *Grillotia* species previously found in the Mediterranean Sea. At intraspecific level, two different genotypes were found. A first type was retrieved only from *D*. *licha*, whereas a second type was found in *G. melastomus*, *E. spinax*, and *S. canicula*.

**Main conclusions:**

Present results suggest that the two genotypes could be involved in different consumer‐resource systems and confirm most of the examined shark species as transport hosts of *Grillotia* species for unknown larger top predators.

## Graphical abstract

This study investigated the factors that determine the occurrence and the level of infection of *Grillotia* (Cestoda) plerocerci in the skeletal muscles of various benthonic sharks and analyzed the parasites through an integrative taxonomic approach. Plerocerci of *Grillotia* were detected in all shark species except *Scyliorhinus stellaris*. Present results suggest that the two genotypes here found could be involved in different consumer‐resource systems and confirm most of the examined shark species as transport hosts of *Grillotia* species for unknown larger top predators.

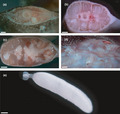

## 1 INTRODUCTION

Cestodes of order Trypanorhyncha Dising, 1863 are among the most common parasites of marine fishes (Palm, 2004). They have a complex life cycle and a unique morphological feature, with both adults and larvae having an anterior extremity with a tentacular apparatus consisting of four retractile tentacles adorned with hooks (Campbell & Beveridge, 1994; Palm, 2004).

Within Trypanorhyncha, the genus *Grillotia* Guiart, 1927 (Family Lacistorhynchidae Guiart, 1927) comprises 18 species infecting the digestive tract of elasmobranchs as definitive hosts (Beveridge & Campbell, 2013; Palm, 2004). Based on the morphological characters of its tentacular apparatus, the genus has been recently divided into two well‐defined species groups (the true *Grillotia* species and those of the subgenus *Christianella* Guiart, 1931) and a third heterogeneous group (the so‐called *Grillotia* *sensu* *lato*) (Beveridge & Campbell, 2007, 2010, 2013).

Four species of *Grillotia*, namely *Grillotia heptanchi* (Vaullegeard, 1899) Dollfus, 1942, *G. adenoplusia* (Pintner, 1903) Palm, 2004, *G. erinaceus* van Beneden, 1858, and *G. scolecina* Rudolphi, 1819 are known from the Mediterranean basin so far (Beveridge & Campbell, 2013; Dallarés, 2016; Paggi, 2008). Among them, *G. heptanchi* and *G. adenoplusia* were found as adults in pelagic sharks, and *G. erinaceus* as adults in skates and rays, and, finally, *G. scolecina* was described using plerocerci collected in *Scyliorhinus stellaris* Linnaeus, 1758 from the Gulf of Naples, but it is now considered a *species inquirenda* in the absence of available type material (see Beveridge & Campbell, 2007, 2010, 2013). All these species but *G. erinaceus* are included in the *Grillotia s*.*l*. species group.

Published literature on the infection of *Grillotia* species in the Mediterranean fishes is scarce, often from the past century, or scattered through fish parasite surveys (Pintner, 1930; Guiart, 1935; Dollfus, 1969; see Beveridge & Campbell, 2013; Dallarés, 2016). Moreover, no molecular data exist from the Mediterranean Sea except for a recent report of a massive infection of plerocerci by an unidentified *Grillotia* species in the anglerfish *Lophius piscatorius* Linnaeus, 1758 (Santoro et al., 2018).

Understanding the factors that determine the occurrence or level of infection in marine fishes has interested scientists for decades and numerous variables about parasite, host, and environment have been identified as important in the dynamics of the host–parasite systems (Dallarés, 2016; Palm, 2004; Santoro, Iaccarino et al., 2020; Timi & Poulin, 2020; Tompkins et al., 2011). In particular, the probability to become infected with larval forms of trypanorhynchans, as well as their levels of infection, depends on a number of biotic and abiotic factors at different geographical scales (Palm, 2004; Palm et al., 2017; Palomba, Santoro et al., 2021; Santoro et al., 2020). The distribution of larval and adult parasites is generally shaped through biotic factors involved in transmission pathways, such as trophic interactions between definitive, intermediate, and transport hosts. For parasites with a life cycle embedded within the marine food webs, biotic factors are also important to evaluate the physiological condition of the fish and the way the fish get resources from the environment, and also the probability for a host to acquire parasites. Therefore, the study of relationships between biotic factors and number of parasites within a host might also provide information about the biology and ecology of both hosts and parasites (Dallares, 2016; Dallares et al., 2016, 2017; Dallares, Padros et al., 2017; Palm, 2004; Santoro, Iaccarino et al., 2020; Timi & Poulin, 2020).

The aim of this study was to characterize through an integrative taxonomic approach the *Grillotia* plerocerci that infect small to mid‐sized benthonic sharks in the Gulf of Naples (Mediterranean Sea) and investigate the features of the infection evaluating how the biological traits of the hosts can influence the occurrence and abundance of the parasites.

## 2 METHODS

### 2.1 Collection and shark examination

**FIGURE 1:**
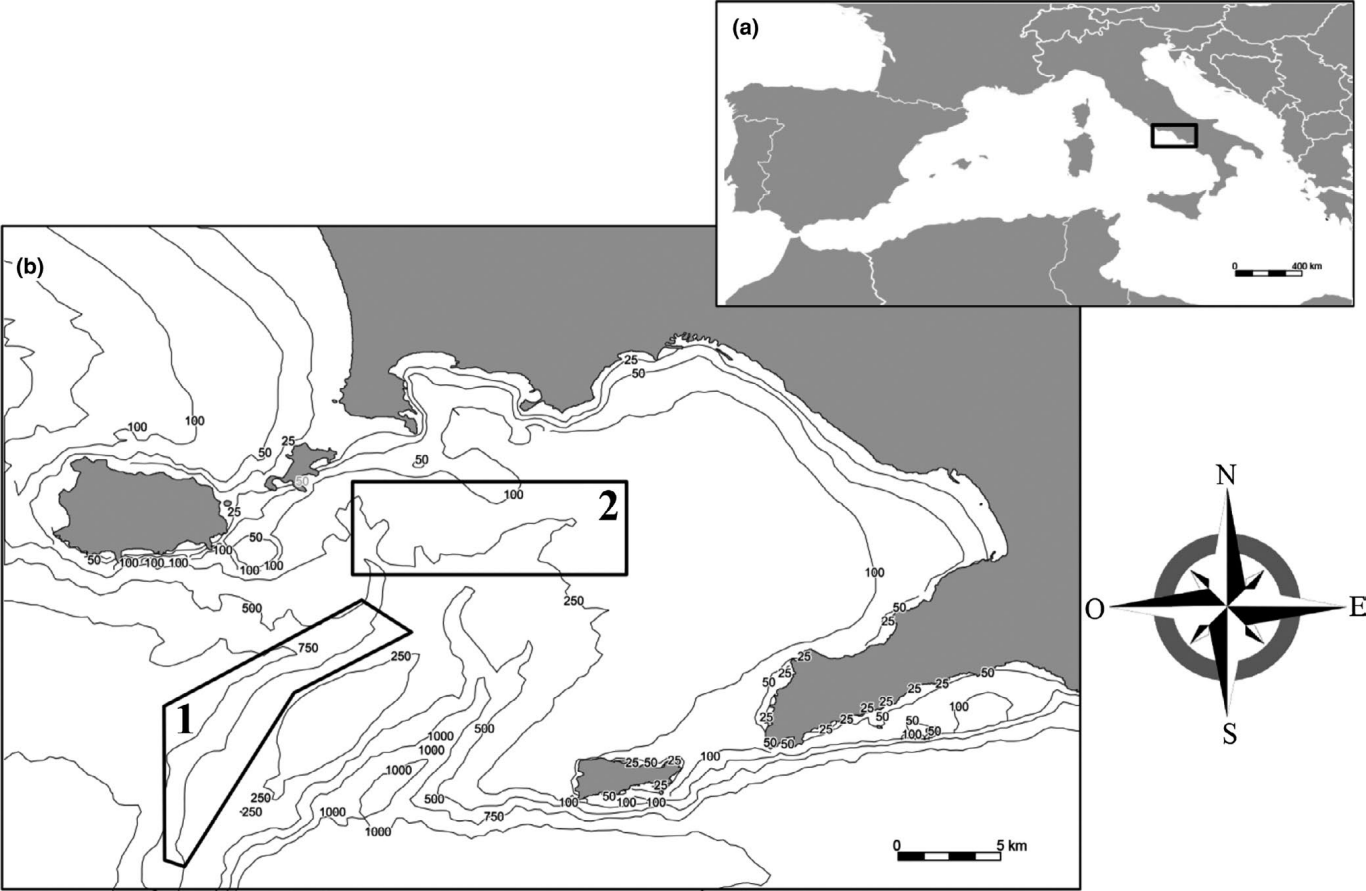
Sampling sites in the Gulf of Naples showing site 1 where all sharks were obtained except individuals of *Scyliorhinus stellaris* which were mostly obtained from the site 2

A total of 245 individuals of five benthic shark species were collected from the Gulf of Naples and frozen until dissections were performed. Sampling included 104 *Scyliorhinus canicula* Linnaeus 1758, 91 *Galeus melastomus* Rafinesque 1810, 39 *Etmopterus spinax* Linnaeus 1758, 8 *Scyliorhinus stellaris*, and 3 *Dalatias licha* Bonnaterre 1788. All specimens were obtained between July and August 2020 in the trawling area between Ischia and Capri Islands (~40.575816N, 13.966513E) at ~400–600 m depth (Figure 1, site 1), except for the specimens of *S*. *stellaris*, that were obtained between July and December 2020 at ~100–400 m depth (Figure 1, site 1 and 2, ~40.729553N, 14.135465E).

Sharks used in the present study constituted the bycatch of scientific and commercial trawling operations (red shrimps’ and pink shrimps’ fishery activities) held with a commercial fishing vessel equipped with bottom trawl nets (mouth of 3 × 4 m in height and width, respectively; 40 mm mesh size), towed at ~2–2.5 kn on muddy bottoms (Crocetta et al., 2020; Palomba et al., 2021). Samplings were performed in accordance with the permit n. 0008453 (issued May 15, 2020) by the Italian Ministry of Agricultural, Food and Forestry Policies, the guide for the care and use of animals by the Italian Ministry of Health and the ARRIVE guidelines.

After thawing, the sharks were weighed to the nearest 0.1 g and measured (fork length‐FL) to nearest 0.1 cm; sex was determined by gonadal examination. A macroscopic gonadal maturity score (GMS) was recorded to investigate the phase of the reproductive cycle (1 = immature, 2 = maturing, 3 = mature, 4 = resting/regressing) as described in Follesa and Carbonara (2019). Body condition index (BCI, whole weight/fork length^3^) was calculated as described by Le Cren (1951). The gonadosomatic (GSI, gonad weight/host eviscerated weight × 100) and hepatosomatic indices (HSI, liver weight/host eviscerated weight × 100) were calculated as suggested by Mouine et al. (2007).

The musculature of each fish was first cut in thin slices (about 0.5 cm in thickness) and examined under a dissecting microscope for trypanorhynchan larvae. Then, each muscle slide was shredded in a Petri dish with purified sea water and all parasites were collected. Parasites attached to the musculature were extracted using scissors and tweezers. Subsequently, all larvae were counted, washed in physiological saline solution, and preserved in 70% ethanol or frozen (−20°C) for morphological and genetic analyses, respectively. Larval forms were clarified in Amman's lactophenol, and the main morphological characters of scolex and bothria were used to identify the larvae at the lowest possible taxonomic level, according to the identification keys proposed by Palm (2004).

For *SEM* analyses, five plerocerci were also fixed overnight in 2.5% glutaraldehyde, then transferred to 40% ethanol (10 min), rinsed in 0.1 M cacodylate buffer, postfixed in 1% OsO_4_ for 2 hr, and dehydrated in ethanol series, critical point dried, and sputter‐coated with platinum. Observations were made using a JEOL JSM 6700F scanning electron microscope operating at 5.0 kV (JEOL, Basiglio, Italy).

Selected samples of skeletal muscles of infected fish were fixed in 10% neutral phosphate‐buffered formalin for histological examination and processed by routine methods into paraffin blocks which were cut into 3‐μm‐thick sections and stained with hematoxylin and eosin.

### 2.2 Molecular analyses for *Grillotia* identification

Due to the high number of larvae retrieved, a representative subsample of 80 *Grillotia* plerocerci, 20 from each positive fish species (*S. canicula*, *G. melastomus*, *E. spinax*, and *D. licha*), was used for molecular analyses. Total genomic DNA (gDNA) from each larva was extracted using the Quick‐gDNA Miniprep Kit (ZYMO RESEARCH), following the standard manufacturer‐recommended protocol.

The partial large subunit (lsrDNA, 28S) was amplified using the primers ZX‐1 (5′ – ACCCGCTGAATTTAAGCATAT – 3′ and 1500R (5′ – GCTATCCTGAGGGAAACTTCG – 3′) (Palm et al., 2009; Van der Auwera et al., 1994). PCR was carried out in a 25 µl volume containing 0.6 µl of each primer 10 mm, 2 µl of MgCl_2_ 25 mm (Promega), 5 µl of 5× buffer (Promega), 0.6 µl of dNTPs 10 mm (Promega), 0.2 µl of Go‐Taq Polymerase (5 U/µl) (Promega), and 2 µl of total DNA. PCR temperature conditions were the following: 94°C for 3 min (initial denaturation), followed by 35 cycles at 94°C for 30 s (denaturation), 53°C for 30 s (annealing), 72°C for 2 min (extension), followed by postamplification at 72°C for 7 min. PCR amplicons were purified using the AMPure XP kit (Beckman Coulter) following the standard manufacturer‐recommended protocol and Sanger sequenced from both strands using the same primers through an Automated Capillary Electrophoresis Sequencer 3730 DNA Analyzer (Applied Biosystems), by the BigDye® Terminator v3.1 Cycle Sequencing Kit (Life Technologies). Contiguous sequences were assembled and edited using MEGA X v. 11 (Kumar et al., 2018). Sequence identity was checked using the Nucleotide Basic Local Alignment Search Tool (BLASTn) (Morgulis et al., 2008). The obtained 28S sequences were aligned with the published sequences of Lacistorhynchidae using ClustalX v. 2.1 (Larkin et al., 2007). JModelTest v. 2.1.10 was used to select the best fit model using the Akaike information criterion (Posada, 2008; Posada & Buckley, 2004). Representative sequences obtained in the present study were deposited in GenBank under the accession numbers MW838227–MW838238.

Bayesian inference (BI) was constructed using MrBayes, v. 3.2.7 (Huelsenbeck & Ronquist, 2001). The Bayesian posterior probability analysis was performed using the MCMC algorithm, with four chains, 0.2 as the temperature of heated chains, 5,000,000 generations, with a subsampling frequency of 500 and a burn‐in fraction of 0.25. Posterior probabilities were estimated and used to assess support for each node. Values with a 0.90 posterior probability were considered well supported. Maximum likelihood (ML) analysis was performed using IQ‐TREE (Nguyen et al., 2015) with 1,000 ultrafast bootstrap replicates. Clades were considered to have high nodal support if the ML bootstrap resampling ≥70%. Due to the phylogenetic position of the family Lacistorhynchidae (see Palm et al., 2009), the phylogenetic tree was rooted using *Nybelinia surmenicola* Okada in Dollfus, 1929 as outgroup. The 28S sequences from GenBank included in the phylogenetic tree are listed in Table 1.

**TABLE 1:** Species, stages (L: larva, A: adult), host, locality, and accession numbers of 28S sequences of the Lacistorhynchidae family included in the phylogenetic analyses shown in the Figure 4

Species	Stage	Host	Locality	28S	References
*Batygrillotia rowei*	L	*Coryphaenoides armatus*	Australia	DQ642765	Olson et al. (2010)
*Callitetrarhynchus gracilis*	L	*Scomberomorus commerson*	Indonesia	FJ572957	Palm et al. (2009)
*Dasyrhynchus variouncinatus*	L	*Caranx sexfasciatus*	Indonesia	FJ572950	Palm et al. (2009)
*Diesingum lomentaceum*	A	*Mustelus mustelus*	Senegal	DQ642760	Olson et al. (2010)
*Floriceps minacanthus*	L	*Euthynnus affinis*	Australia	AF286971	Olson et al. (2001)
*Floriceps saccatus*	L	*Lepidocybium flavobrunneum*	Indonesia	FJ572958	Palm et al. (2009)
*Grillotia erinaceus*	A	*Raja radiata*	United Kingdom	AF286967	Olson et al. (2001)
*Grillotia pristiophori*	A	*Pristiophorus nudipinnis*	Australia	DQ642763	Olson et al. (2010)
*Grillotia yuniariae*	L	unidentified species of *Ophiidae*	Indonesia	FJ572952	Palm et al. (2009)
*Grillotia sasciae*	A	*Raja straeleni*	South Africa	MW081569	Oosthuizen et al. (2021)
*Grillotia* sp.	L	*Lophius piscatorius*	Italy (Tyrrhenian Sea)	MH664054	Santoro et al. (2018)
*Grillotia* sp. 1	A	*Amblyraja radiata*	Canada: Bay of Fundy	MH688700	Beer et al. (2018)
*Grillotia* sp. 2	A	*Bathyraja brachyurops*	Falkland Islands	MH688707	Beer et al. (2018)
*Grillotia* sp. 3	A	*Bathyraja magellanica*	Falkland Islands	MH688704	Beer et al. (2018)
*Grillotiella exile*	L	*Scomberomorus commerson*	Indonesia	FJ572953	Palm et al. (2009)
*Hornelliella annandalei*	A	*Stegostoma fasciatum*	Indonesia	FJ572956	Palm et al. (2009)
*Lacistorhynchus dollfusi*	A	*Mustelus antarcticus*	Australia	DQ642761	Olson et al. (2010)
*Lacistorhynchus tenuis*	A	*Mustelus canis*	USA, NE Atlantic	FJ572955	Palm et al. (2009)
*Nybelinia surmenicola*	L	*Peurogrammus azonus*	Japan	FJ572929	Palm et al. (2009)
*Paragrillotia similis*	A	*Ginglymostoma cirratum*	USA, Florida	FJ572954	Palm et al. (2009)
*Pseudogrillotia* sp.	A	*Carcharhinus amboinensis*	Australia	DQ642767	Olson et al. (2010)
*Pseudogilquinia microbothria*	A	*Sphyrna mokarran*	Australia	DQ642766	Olson et al. (2010)
*Pseudogilquinia pillersi*	L	*Lethrinus atkinsoni*	Australia	AF286964	Olson et al. (2001)
*Pseudolacistorhynchus heroniensis*	L	*Pectropomus leopardus*	Australia	AF286968	Olson et al. (2001)

Genetic distances (shown as the percentage difference, i.e., no. of base substitutions per sites *100) were computed using the Kimura 2‐Parameters (K2P) model (Kimura, 1980) with 1,000 bootstrap resamplings, by MEGA Software, version 7.0.

### 2.3 Statistical analyses

Prevalence and abundance of infection used in the present study follow Bush et al. (1997). Only host species with a sufficient number of individuals including *E. spinax*, *G. melastomus*, and *S*. *canicula* were used for statistical analyses. Differences in the abundance of *Grillotia* in the three shark species were measured by means of the nonparametric rank‐based Mann–Whitney *U* test.

Boosted regression trees (BRTs) were then used to model the relationship between the number of *Grillotia* specimens found in each host with both morphological (FL, weight, and BCI) and physiological predictors (age, sex, SMS, GSI, and HSI) (Santoro, Iaccarino et al., 2020). BRTs are a class of nonparametric machine learning method where regression trees are combined in an ensemble boosting algorithm to improve predictive performance. Cross‐validation (with max trees = 10,000) was used to find the optimal settings in terms of model parametrization and to avoid overfitting due to the relatively low number of specimens in our samples (De’Ath, 2007; Elith et al., 2008; Leathwick et al., 2006). Models were therefore trained by using values of learning rate (*lr*, the contribution of each regression tree to the model) of 0.01 and tree complexity (*tr*, model complexity in terms of allowed interactions among predictors) of 5, with bag fraction values (i.e., the proportion of observations used in selecting variables) of 0.5 and assuming a Gaussian error distribution.

Contributions of predictors were expressed by their relative influence (Friedman & Meulman, 2003), scaled in a 0–100% scale where higher percentage indicates a stronger influence on the response variable and can thus be considered more influential. Partial dependence plots were used to model the effect of predictors on the response variable after accounting for the average effects of all other variables in the model (Friedman, 2001; Friedman & Meulman, 2003). Model performance was measured by using the explained deviance, computed as 1‐(mean residual deviance/mean total deviance) and with values of 1 indicating a perfect fit. Model accuracy was assessed by means of the Spearman's rank correlation values between fitted and observed values. Finally, the *H*‐statistic was used to measure the strength of interaction effects between predictors in BRT models, ranging between 0 and 1 and with higher values corresponding to larger interaction effects. Analyses were performed in R (R Development Core Team, 2018) using the packages *gbm* and *dismo* (Hijmans et al., 2013; Ridgeway et al., 2017) and following the prescriptions in Elith et al. (2008) and Elith and Leathwick (2017).

## 3 RESULTS

### 3.1 General data

**FIGURE 2:**
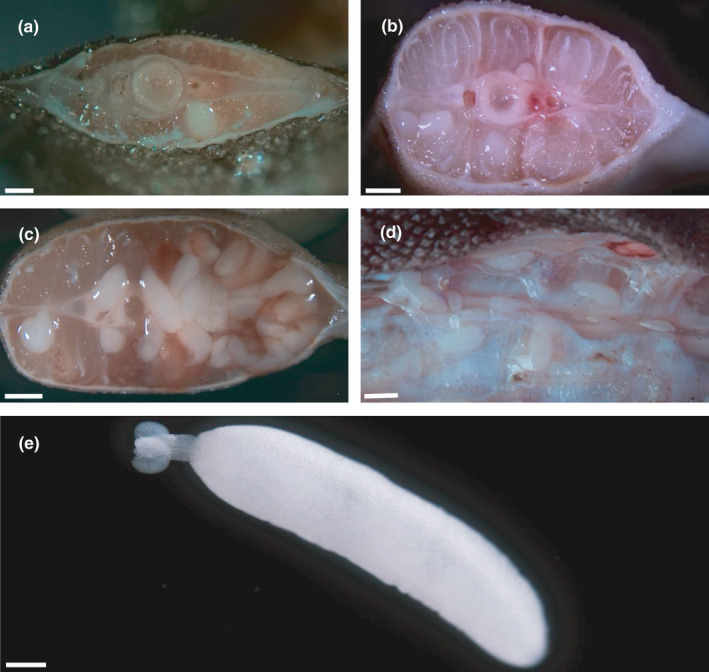
Macrophotographs of skeletal muscles of sharks containing plerocerci of *Grillotia* sp. (a: *E*. *spinax*, b: *S*. *canicula*, c: *G*. *melastomus*, d: *D*. *licha*), and microphotograph of the general view of a plerocercus of *Grillotia* sp. (e) from skeletal muscles of *S*. *canicula*. Scale bar: (a) 1,000 µm; (b c and d) 2,000 µm; (e) 500 µm

**FIGURE 3:**
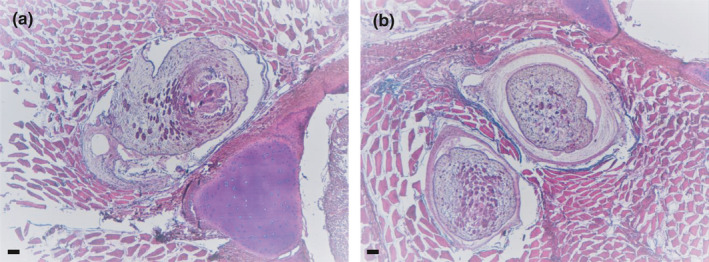
Histological sections (H&E) of skeletal muscles of *S. canicula* (a) and *G. melastomus* (b) showing encapsulated plerocerci of *Grillotia* sp. close to the vertebral column. Note the atrophy of muscle fibers and the scarce inflammatory response. Scale bar: 100 µm

**FIGURE 4:**
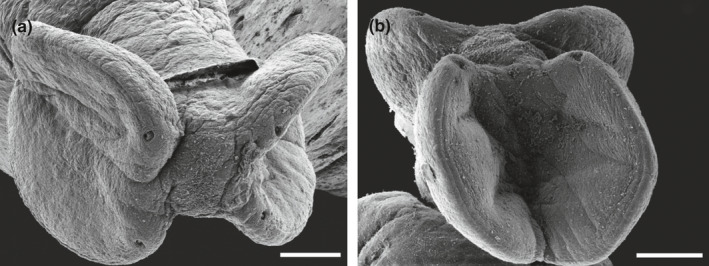
Scanning electron micrographs of anterior extremity of *Grillotia* sp. from the skeletal muscles of *D. licha* (a) and *S. canicula* (b). Note the four openings of the uneverted tentacular apparatus located on the anterior margin of the bothria. Scale bar: 100 µm

Numbers of examined hosts, biometrical data, physiological indices, and basic values of infection for all host species examined are listed in Table 2. Encapsulated plerocerci of *Grillotia* were found in the musculature of all shark species examined except *S. stellaris* (Figure 2). The plerocerci, all identical one each other, were translucent and white in color and ovoid in shape (Figure 2e). They ranged from 4 to 5 mm in length and from 0.9 to 1.2 mm in width (*n* = 20). The plerocerci occurred throughout the muscle tissue close to the vertebral column along the last third of the body shark. Morphological identification to species level was not possible because no plerocerci were found with everted tentacular apparatus. Histological analysis revealed ovoid well‐defined encysted parasites embedded in the muscular tissue close to the vertebral column. The larva was surrounded by edema with compression and atrophy of muscle fibers (Figure 3) and scarce inflammatory response. *SEM* micrographs of the anterior extremity of plerocerci revealed the typical features of genus *Grillotia* with two patelliform bothria in opposite arrangement without thickened rims and bothrial pits absent (Figure 4).

**TABLE 2:** Number, biometrical data, physiological indices, and basic levels of infection in five species of sharks examined for *Grillotia* larvae from the Gulf of Naples

	*D. licha*	*E. spinax*	*G. melastomus*	*S. canicula*	*S. stellaris*
*n*	3	39	91	104	8
Sex	3f	23f, 16m	48f, 43m	52f, 52m	7f, 1m
Fork length (cm)	97.667 ± 5.773 (91–101)	26.651 ± 4.919 (14–36.6)	41.263 ± 7.081 (23.5–56)	39.725 ± 6.652 (14–49.5)	60.271 ± 18.095 (33–80)
Weight (g)	6,113 ± 964.076 (5142–7070)	82.333 ± 40.779 (11–180)	195.549 ± 88.462 (39–369)	233.404 ± 177.231 (6–1746)	1,514.74 ± 1,158.41 (180–2855)
BCI	0.006 ± 0.0005 (0.0068–0.005)	0.004 ± 0.001 (0.002–0.05)	0.003 ± 0.0001 (0.0003–0.004)	0.003 ± 0.025 (0.002–0.028)	0.005 ± 0.0009 (0.0045–0.0067)
HSI	0.496 ± 0.075 (0.413–0.595)	0.161 ± 0.062 (0.047–0.269)	0.036 ± 0.02 (0.0103–0.095)	0.074 ± 0.031 (0.021–0.172)	0.117 ± 0.036 (0.076–0.179)
GSI	0.308 ± 0.223 (0.062–0.499)	0.004 ± 0.006 (0–0.028)	0.026 ± 0.03 (0–0.138)	0.042 ± 0.044 (0–0.164)	0.0303 ± 0.037 (0–0.086)
Prevalence %	100	82.05	100	94.23	0
Abundance	169.33 ± 184.66 (27–378)	5.33 ± 6.03 (1–27)	181.65 ± 189.13 (17–1421)	32.99 ± 30.77 (1–188)	0

Note

Values are expressed as mean ± standard deviation with range in parenthesis.

Abbreviations: BCI, body condition index; f, female; GSI, gonadosomatic index; HIS, hepatosomatic index; m, male.

### 3.2 Molecular identification of *Grillotia* plerocerci

The BLASTn analysis of the 80 sequences of *Grillotia* obtained at the 28S gene locus (1,450 bp) retrieved a percentage of identity of ∼99.40% (E‐value = 0.00) with a partial sequence of *Grillotia* sp. (MH664054) (481 bp) previously found in an anglerfish from Tyrrhenian Sea by Santoro et al. (2018). At intraspecific level, two genotypes were found. Sequences of *Grillotia* specimens from *D. licha* were identical to each other; however, these differed from those obtained from the other shark species (*S. canicula*, *G. melastomus*, and *E. spinax*) by 0.22%. Sequences from *D. licha* differed in 3 nucleotide sites, showing a G instead of an A at the position 731, an A instead of a G at the position 869, and a G instead of an A at the position 1,184. *Grillotia* sequences from *G. melastomus*, *E. spinax*, and *S. canicula* at the same gene locus were identical.

**FIGURE 5:**
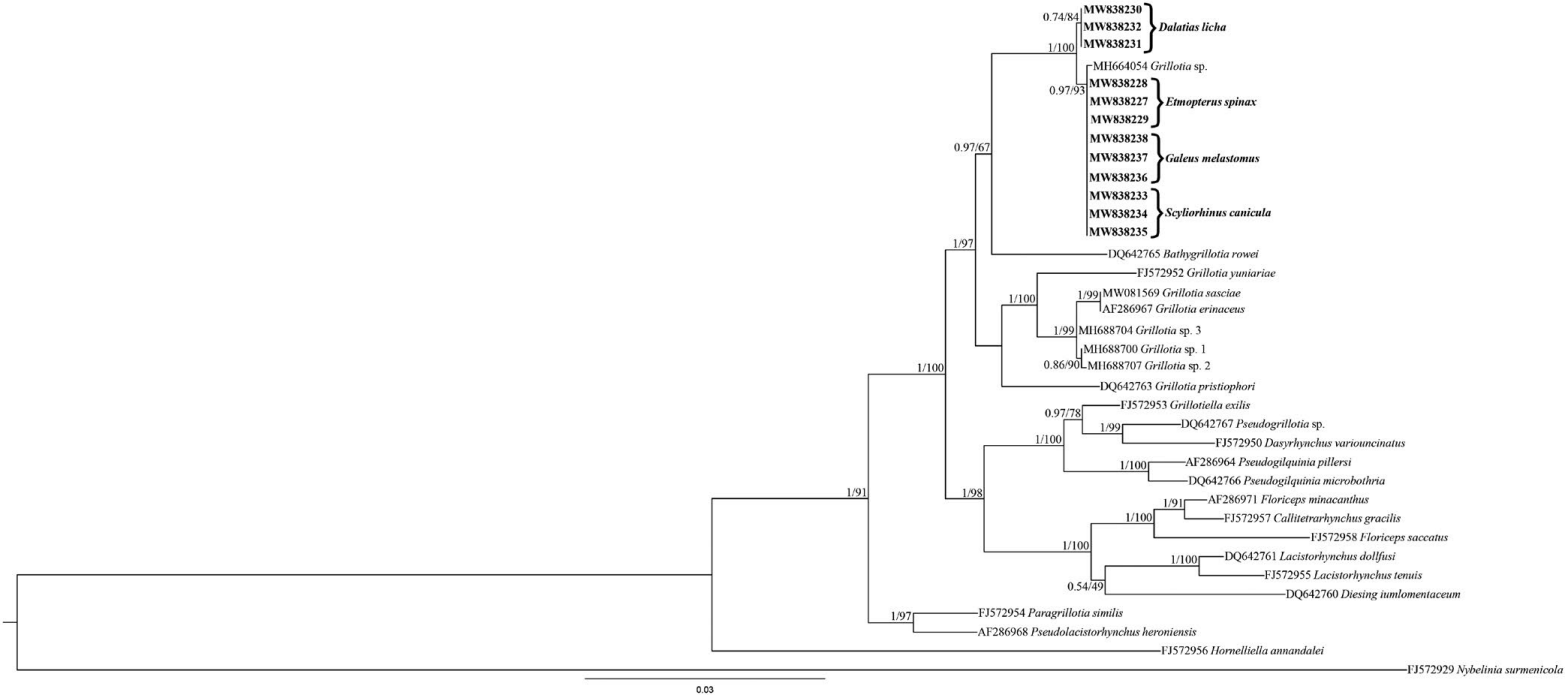
Phylogenetic tree from Bayesian Inference (BI) and maximum likelihood (ML) based on 28S sequences of the specimens of *Grillotia* sp. obtained in the present study (in bold), with respect to the sequences of the same genus and other genera of the family Lacistorhynchidae available in GenBank. The analysis was performed by MrBayes v. 3.2.7 and IQ‐TREE, using the TIM3 + I + G substitution model, as implemented in jModeltest 2.1.10. Nodal support is indicated for BI (posterior probabilities) and ML (bootstrap, *n* = 100), respectively. *Nybelinia surmenicola* was used as outgroup

The BI and ML tree topologies as inferred from the phylogenetic analysis showed that the 28S sequences clustered in a single clade, which also includes the sequence MH664054 deposited by Santoro et al. (2018). However, the two genotypes obtained and the sequence of *Grillotia* species already available in GenBank clustered in two separate branches, too (Figure 5). The distance value between the sequences obtained here and the MH664054 sequence was 0.19%. The sequences included in this clade showed a close relationship with the sequence of *Bathygrillotia rowei* (Campbell, 1977) Beveridge & Campbell, 2012 (syn. *G. rowei*) retrievable in GenBank (DQ642765). Indeed, the distance values between the present *Grillotia* specimens and *B*. *rowei* resulted to be 3% and 3.1%, thus not guarantying conspecificity.

### 3.3 Statistical analysis

Shark species significantly differed in terms of *Grillotia* abundance (Mann–Whitney *U* test, n_spinax_ = 39, n_melastomus_ = 91, n_canicula_ = 104, U > 710, *p* < .001 in all pairwise tests), with *G*. *melastomus* being characterized by the highest number of parasites, followed by *S*. *canicula* and *E*. *spinax* (Table 2).

**FIGURE 6:**
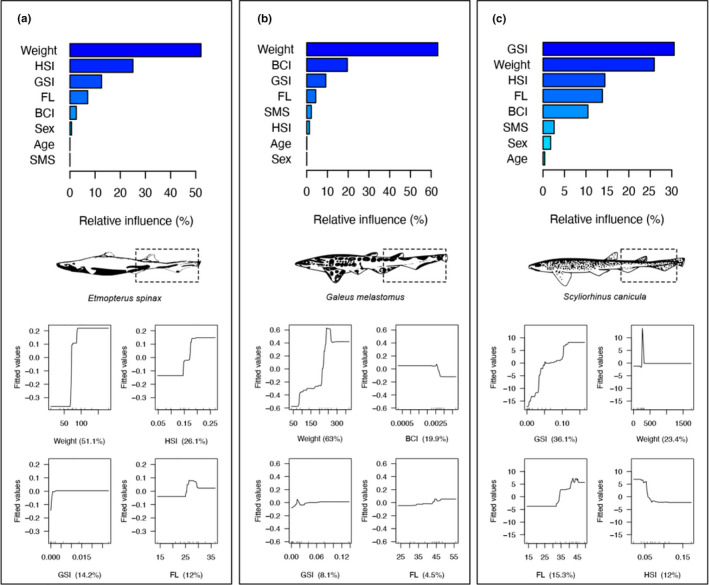
Results of the boosted regression tree models showing the relative influence (in percentage) of morphological (FL, weight, and BCI) and physiological predictors (age, sex, SMS, GSI, and HSI) on the number of *Grillotia* specimens found in each host species. BCI, body condition index; HIS, hepatosomatic index; FL, fork length; GSI, gonadosomatic index; SMS, sex maturity score. Sharks in the figure have been redrawn from Compagno (1984a, 1984b) and downloaded from https://fishbase.org under a Creative Commons License—CC BY‐NC 3.0. Dotted boxes on shark tails show approximately the body sites where *Grillotia* specimens have been collected

Boosted regression trees models were able to accurately predict the abundance of *Grillotia* in all shark species having on average a higher prediction accuracy in *G*. *melastomus* and *S*. *canicula* than in *E*. *spinax*. Weight, HSI, GSI, FL, and BCI were the most important predictors in all species, while traits related to sex, SMS, and age were less reliable predictors accounting for a very limited percentage (Figure 6). Although the traits involved in explaining the abundance of *Grillotia* were similar for all species, their relative influence significantly differed among species. Traits more related to size (e.g., weight and, to a lesser extent, BCI) had a relatively high contribution in *G*. *melastomus*, while both morphology‐ and physiology‐related traits (e.g., GSI and HSI) mostly explained the observed pattern in *E*. *spinax* and *S*. *canicula* (Figure 6). For instance, weight and HSI accounted for ca. 80% in *E*. *spinax*, showing a step‐like relationship characterized by a sudden increase for weight values between ca. 65–100 g and HSI values between ca 0.15 and 0.20, followed by a *plateau* (Figure 6a). These results suggest that medium‐weighted individuals with intermediate values of HSI in *E*. *spinax* would be subjected to a high infection rate, while larger individuals having higher HSI values would not show a substantial increase in the number of *Grillotia* hosted (Figure 6a).

In *G*. *melastomus*, weight accounted for more than 60%, having a positive relationship with the abundance of *Grillotia,* while BCI accounted only for ca. 19.9% (Figure 6b). In *S*. *canicula*, GSI and weight accounted for more than 50%, observing an increasing of *Grillotia* for increasing GSI values and a peak in a window of values restricted to individuals of low weight, followed by a plateau on values similar to those observed before the peak (Figure 6c).

The most important predictors of *Grillotia* in *G*. *melastomus* and *S*. *canicula* produced relatively strong interaction effects, while for *E*. *spinax* this did not occur (Figure 6). In particular, weight in *G*. *melastomus* had a relatively strong interaction effect with both BCI and GSI (*H*‐statistics 0.19 and 0.13, respectively) and only a marginal effect with FL (*H*‐statistics 0.05). In *S*. *canicula*, GSI had a strong interaction with weight (*H*‐statistics 0.21) but weak interactions with both FL and HSI (*H*‐statistics 0.12 and 0.11, respectively). The magnitude of interaction effects in *E*. *spinax* was very weak for each combination of the most important factors (*H*‐statistics <10^–15^), a situation likely to be related to the low number of individuals which might have contributed also to the low accuracy of BRTs in this species.

## 4 DISCUSSION

According to the GenBank database, 28S rDNA molecular data exist only from three (*G. yuniariae*, *G. erinaceus*, and *G. pristiophori*) out of the 18 species of *Grillotia* known at present, with a single 28S rDNA sequence available for an unidentified *Grillotia* species from the Mediterranean basin (Santoro et al., 2018). Molecular comparisons of the present plerocerci with data retrieved from GenBank confirmed that the present material did not match with the three above mentioned species. Moreover, sequence comparisons between plerocerci from *D. licha* and the other three shark species (*E. spinax*, *G. melastomus*, and *S. canicula*) documented differences which provided support for the existence of two potential genotypes, each of which could have a different definitive host and could be involved in different food web systems. In fact, whether *D. licha* preyed on small sharks (e.g., *G. melastomus* and *S. canicula*) as suggested by previous studies in Spanish waters (Matallanas, 1982; Navarro et al., 2014), it would show at least a mixed infection with both genotypes of *Grillotia* sp. found in the present study. In support of our hypothesis, it has been suggested that parasites, in the process of host switch adaptation, may produce different genotypes that involve substitutions on a small number of nucleotides. Such minor genetic modifications can then become associated with different host species (Poulin et al., 2011; Woolhouse et al., 2001).

Life cycle proposed for members of *Grillotia* includes three/four hosts, with copepods as first, schooling fishes as second, and larger predatory fishes as third intermediate hosts, with species of elasmobranchs acting as their definitive hosts (Beveridge & Campbell, 2007; Lubieniecki, 1976; Palm, 2004). As an example, *G*. *erinaceus*, which mature in skates, uses calanoid copepods as first intermediate hosts and encyst in the viscera of fishes as second intermediate hosts. Skates becomes finally infected by preying on those fishes (Lubieniecki, 1976; Palm, 2004). An association between *G. erinaceus* and the Mediterranean teleost bigeye rockling *Gaidropsarus biscayensis* Collett, 1890 has been reported by Dallarés et al. (2016), suggesting that the infection of benthopelagic predatory fish with plerocerci of *Grillotia* may occur through the ingestion of fishes which feed on infected copepods. Plerocerci of *Grillotia* morphologically identified as *G. adenoplusia* (a species which mature in pelagic sharks) were common in skeletal muscles of *Ga*. *melastomus* from the North‐Western Mediterranean coast of Spain, where the infection was linked to the consumption of myctophid fish, which in turn are known to prey on copepods (Dallarés, 2016). In the present study, we did not focus the attention on the quantification and identification to the lowest taxonomic level of the gastrointestinal contents. However, gross analysis of gastrointestinal material during the parasitological analysis revealed that remains of myctophid fishes and cephalopods were among the most common food items found in *E. spinax*, *G. melastomus*, and *S. canicula*, supporting a role of these prey as possible transport hosts for *Grillotia* specimens.

In Mediterranean Sea, recent records of larval forms of *Grillotia* infecting sharks include two reports in *G. melastomus*, in which the larvae were originally reported as *Grillotia* sp. (Dallares, Padros et al., 2017) and then as *G*. *adenoplusia* (Dallares et al., 2017), with a prevalence of infection ranging from 7.6% to 28.6% and a maximum mean abundance of 0.48 depending from the sampling localities, whereas no *Grillotia* specimens were detected in 41 individuals of *S. canicula* from the same locality (Dallares, Padros et al., 2017; Dallares et al., 2017). The difference in infection values between the North‐Western Mediterranean studies (Dallares, Padros et al., 2017; Dallares et al., 2017) and the present one might be related to the different geographical features and high productivity in terms of food webs at the time of the sampling, two variables that may affect the number of intermediate and definitive hosts present in the area (Collins et al., 1984; Dallares, Padros et al., 2017; Hassan et al., 2002; Palm & Overstreet, 2000; Santoro et al., 2019; Santoro, Iaccarino et al., 2020).

The habitat use and the feeding ecology of the hosts might explain some results obtained in the present study (Jakob & Palm, 2006; Palm & Caira, 2008; Palm et al., 2007), among which: (i) the absence of *Grillotia* plerocerci in *S. stellaris*, which mostly use different habitat and prey than other shark species examined in the present study; (ii) the occurrence of the same *Grillotia* genotype in the three sympatric shark species with similar feeding habit (*E. spinax, G. melastomus*, and *S. canicula*); and finally, (iii) the occurrence of a different genotype in *D. licha* which has different feeding habit than the previous three species.

Dallares, Padros et al. (2017) suggested *D. licha*, or alternatively the bluntnose sixgill shark *Hexanchus griseus* Bonnaterre 1788, as definitive hosts for the specimens of *Grillotia* found in *G. melastomus* in the North‐Western Mediterranean, as both former species mostly feed on small sharks. Unfortunately, no sequences of specimens of *Grillotia* identified by Dallares, Padros et al. (2017) and Dallares et al. (2017) are so far available in GenBank for comparison with the present sequences.

We think that a member of the genus *Hexanchus* Rafinesque, 1810 (i.e., *H. griseus* and/or *Hexanchus nakamurai* Teng, 1962) could be among the definitive hosts for at least a genotype of *Grillotia* here found, being the only benthic big sharks capable to feed on small to mid‐sized deep sea benthic sharks in the study area. This could also explain why the larvae were all found in the muscle of the host tail. It has been suggested that different trypanorhynchans evolved different life strategies for completing their biological cycle likely as a result of host–parasite coadaptation and coevolutionary processes (Palm, 2004; Palm et al., 2007, 2009, 2017). The hunting strategy followed by the bluntnose sixgill shark is a tail‐on approach preying from behind, which would presumably enhance the transmission of parasites concentrated in the tail region (Dallarés, 2016; Santoro et al., 2018; Seamone et al., 2014).

Several authors included either the length or the weight of the fish as an explanatory factor for trypanorhynch distributions with strong positive correlation between fish size and trypanorhynch abundance or intensity (see Palm, 2004). In the present study, the weight of the host was the most important predictor for the overall parasite abundance in *G. melastomus* and *E. spinax* and the second most important (after GSI) in *S. canicula*, while the length of the host alone showed negligible importance in all host species, with BRT values ranging from 4.8% to 12.9% (Figure 6). Moreover, in *G. melastomus*, also the BCI (based on length and weight relationships) was a discrete predictor of abundance (18.8%) (Figure 6b), suggesting that in general body size is an important predictor for the overall parasite abundance.

Assuming that fish size increases with age, larger and older fishes are expected to accumulate in muscles a larger number of larval forms of parasites with long life span. MacKenzie (1985, 1987) observed that the plerocerci of *Christianella minuta* (Van Beneden, 1849) Campbell & Beveridge, 1994 (syn. *Grillotia angeli* Dollfus, 1969), encysted in the visceral cavities of mackerels, may live for at least 10 years and accumulate throughout the entire life span of the host. Palm and Overstreet (2000) reported that the intensity of infection with plerocerci of *Otobothrium cysticum* Mayer, 1842 (a species closely related to Lacistorhynchinae and Grillotiinae) increases with host weight in muscles of the butterfish *Peprilus burti* Fowler, 1944 and of the harvestfish *P. paru* Linnaeus, 1758 (syn. *P. alepidotus* Linnaeus, 1766), suggesting a continual accumulation of the larvae in association with little host resistance. The increase in intensity of infection of larvae of *Poecilancistrium caryophyllus* (Diesing, 1850) Dollfus, 1929 with size of the host found in the spot *Leiostomus xanthurus* Lacepède, 1802 and the spotted seatrout *Cynoscion nebulosus* Cuvier, 1830 also might indicate that there may not be an immune response of the host to the larval parasites in muscles, being large number of larvae well tolerated and capable to accumulate in the host musculature over time (Collins et al., 1984). Results described in the present study agree with data from previous studies also regarding the scarce immune response in presence of large amounts of larvae (the maximum number of larvae found was 1421 in an adult male *G. melastomus*), suggesting that the present lesions are probably the result of direct tissue damage rather than an immune reaction targeted toward the parasitic antigens.

Present results support the hypothesis that larger fishes, having in general a higher food intake, consume more infected preys and have greater prevalence and abundance of food‐transmitted parasites, and that the influence of host size on parasite abundance may apply especially to long‐living parasite larvae. Moreover, larger hosts might better tolerate high parasite loads in terms of body mass, offering more space for parasite distribution, and this may be especially relevant for larval forms of trypanorhynchans which encyst in skeletal musculature (Collins et al., 1984; Hassan et al., 2002; Palm, 2004; Palm & Overstreet, 2000). All these considerations, and in particular the smaller body mass, might also explain why *E. spinax* has lower abundance of infection.

Among the indices for evaluating the physiological condition of the fishes (GSI, HIS, and SMS), only GSI was a discrete predictor in *S. canicula*, with a value of 29.9%, while HSI (14.2%) showed lower relative importance (Figure 6c). GSI is a good indicator of reproductive activity of fish, while the HSI is strictly related to the gonadal maturation and in general increases at early ripening and decreases at late ripening (Rizzo & Bazzoli, 2020). In the present study, most of the specimens of *S. canicula* were in reproductive activity (70.2%) suggesting that this physiological condition can modify the immune responsiveness increasing susceptibility to parasitic infections due to an increase in circulating levels of sex steroid hormones. For example, at time of reproductive activity, salmonid fish demonstrate immune deficiencies that include an inability to produce isohaemagglutinins that thus make fishes more susceptible to the ectoparasitic infestations (Pickering & Christie, 1980). Alternatively, it is also plausible that shark individuals in reproductive activity feed more acquiring more parasite individuals.

## 5 CONCLUSION

In conclusion, this study describes the first molecular characterization of larval forms of *Grillotia* in sharks from the Mediterranean Sea, infecting multiple hosts with potential evidence of the development of host specific lineages. According to the host–parasite list of the Shark‐References Database (https://shark‐references.com/species/host‐parasites‐list) and formerly published studies, this represents the first report of *Grillotia* specimens in *E. spinax* and *S. canicula*, and the first time that this larval form is found in *D. licha*. This is also the first contribution to the study of factors which may influence the occurrence and abundance of *Grillotia* specimens in sharks. Finally, the common occurrence of *Grillotia*, showing high prevalence and abundance among four species of small to mid‐sized deep sea benthonic sharks in the Gulf of Naples, enlarges its range of distribution, confirming its wide presence in the deep bottoms of the Tyrrhenian Sea, and indicates that these shark species act as transport hosts of *Grillotia* species for larger top predators.

## CONFLICT OF INTEREST

The authors report no conflicts of interest.

## AUTHOR CONTRIBUTION

**Mario Santoro:** Conceptualization (equal); Data curation (equal); Formal analysis (equal); Funding acquisition (equal); Investigation (equal); Methodology (equal); Project administration (equal); Supervision (equal); Validation (equal); Visualization (equal); Writing‐original draft (equal); Writing‐review & editing (equal). **Bruno Bellisario:** Formal analysis (equal); Writing‐review & editing (equal). **Fabio Crocetta:** Resources (equal); Writing‐review & editing (equal). **Barbara Degli Uberti:** Formal analysis (equal). **Marialetizia Palomba:** Formal analysis (equal).

## Data Availability

28S rDNA sequences: GenBank accessions (MW838227‐MW838238). Final DNA sequence assembly available in the Dryad Digital Repository (at https://doi.org/10.5061/dryad.3n5tb2rh3).
